# A Lightweight Deep Learning Network with an Optimized Attention Module for Aluminum Surface Defect Detection

**DOI:** 10.3390/s24237691

**Published:** 2024-11-30

**Authors:** Yizhe Li, Yidong Xie, Hu He

**Affiliations:** State Key Laboratory of Precision Manufacturing for Extreme Service Performance, College of Mechanical and Electrical Engineering, Central South University, Changsha 410083, China

**Keywords:** image sensors, deep learning network, defect detection

## Abstract

Aluminum is extensively utilized in the aerospace, aviation, automotive, and other industries. The presence of surface defects on aluminum has a significant impact on product quality. However, traditional detection methods fail to meet the efficiency and accuracy requirements of industrial practices. In this study, we propose an innovative aluminum surface defect detection method based on an optimized two-stage Faster R-CNN network. A 2D camera serves as the image sensor, capturing high-resolution images in real time. Optimized lighting and focus ensure that defect features are clearly visible. After preprocessing, the images are fed into a deep learning network incorporated with a multi-scale feature pyramid structure, which effectively enhances defect recognition accuracy by integrating high-level semantic information with location details. Additionally, we introduced an optimized Convolutional Block Attention Module (CBAM) to further enhance network efficiency. Furthermore, we employed the genetic K-means algorithm to optimize prior region selection, and a lightweight Ghost model to reduce network complexity by 14.3%, demonstrating the superior performance of the Ghost model in terms of loss function optimization during training and validation as well as in terms of detection accuracy, speed, and stability. The network was trained on a dataset of 3200 images captured by the image sensor, split in an 8:1:1 ratio for training, validation, and testing, respectively. The experimental results show a mean Average Precision (mAP) of 94.25%, with individual Average Precision (AP) values exceeding 80%, meeting industrial standards for defect detection.

## 1. Introduction

Aluminum products are widely used in many industries including aerospace [1], construction and transportation [2], national defense, and the chemical industry [3], due to their versatility and favorable material properties. However, during production, transportation, and installation, aluminum surfaces may develop defects such as splices, corner leakages, and pits, which significantly impact product quality and reliability. Ensuring that aluminum products meet quality standards is essential to avoid potential failures in critical applications. Thus, effective defect detection has become a crucial step in the manufacturing process, especially in sectors where aluminum integrity directly affects operational safety and performance [4].

Traditional aluminum quality inspection relies predominantly on manual visual inspection and physical testing methods [5], which is labor-intensive and time-consuming. These methods, while straightforward, are highly dependent on the skill and experience of human inspectors, resulting in inconsistent quality assessments. Additionally, physical testing can be destructive and time-consuming, which adds substantial labor costs and limits throughput [6]. Furthermore, manual and traditional methods struggle to detect subtle or complex defects and are generally inefficient for large-scale industrial applications [7]. As a result, there is a growing need to develop automated, non-destructive, and high-precision detection methods to meet the demands of modern manufacturing and quality control processes.

Machine vision and deep learning-based defect detection methods have recently gained attention for their ability to improve efficiency and accuracy over traditional methods. Deep learning models, particularly convolutional neural networks (CNNs), have demonstrated strong capabilities in image-based defect detection due to their ability to learn and represent complex features within images [8]. By leveraging large datasets and computational power, deep learning models can automatically extract and analyze image features, making them particularly suitable for detecting subtle defects that are difficult to identify through traditional methods. Moreover, CNNs enable continuous improvement as they are retrained with new data, making them adaptable to varying defect patterns in aluminum surfaces. Recent advancements in deep learning architectures have led to several notable contributions to aluminum defect detection. Lin et al. proposed a top-down feature pyramid network (FPN) structure, which constructs high-level semantic feature maps across multiple scales, and has shown significant progress as a general feature extractor in detection applications [9]. Li et al. utilized the Faster R-CNN network for aluminum surface defect detection, combining the feature pyramid network with the backbone network ResNet 50 to enhance the utilization of low-level position information and reduce information loss during the convolution process, thereby improving detection efficiency [10]. Building on this approach, Xiang et al. replaced the rough ROI-Pooling algorithm with ROI-Align, enabling more precise defect localization [11]. Additionally, Ma et al. used the Faster R-CNN with a ResNet-101 backbone to enhance the model’s capability to detect large-scale defects by fusing the feature pyramid structure [12].

Despite these improvements, the variety of surface defects and detection noise continue to hinder the accuracy of defect detection methods presented in the literature. For instance, deformable feature pyramids, as proposed by Wang et al., allow the fusion of deep and low-level features, but their approach may still be sensitive to noise in images, which impacts detection accuracy [13]. Additionally, Woo et al. introduced the Convolutional Block Attention Module (CBAM) to enhance CNN models’ feature extraction capabilities, yet CBAM’s effectiveness under noisy conditions is limited [14]. Cao et al. incorporated the attention mechanism into neural networks to detect aluminum surface pits, enabling better multi-task feature fusion, but noise in production environments still affects detection reliability [15]. Han et al. proposed a Ghost module to build a lightweight CNN network structure, but reducing computational cost also risks sacrificing feature detail [16].

However, the diversity of surface defects and the presence of detection noise severely affect the accuracy of methods presented in the literature. Additionally, the training and learning efficiency of current networks has yet to fulfill the detection requirements in real-world applications. To address these challenges, we propose a method based on a modified two-stage Faster R-CNN network, designed for accurate detection of various aluminum surface defects, including variegation, lapel, orange peel, under screen, abrasion, non-conductive spots, pits, and slugs. Specifically, we adopted a multi-scale feature pyramid to fuse high-level semantic information with location details, enhancing recognition accuracy. Additionally, a hybrid attention module was introduced to improve convolutional efficiency, thereby increasing detection precision. An optimized Convolutional Block Attention Module (CBAM) was further employed to reduce the model size, and a genetic K-means unsupervised algorithm, which was chosen for its ability to efficiently explore the solution space and avoid local optima during clustering, was used to optimize prior frame selection, as K-means has consistently demonstrated superior performance in clustering tasks, enhancing the accuracy of defect localization. Finally, the Ghost model was applied to create a lightweight network, reducing model parameters and accelerating training speed. This comprehensive approach effectively addresses the limitations of current methods and achieves robust and efficient defect detection.

## 2. Proposed Deep Learning Network

### 2.1. The Deep Learning Network Framework

Faster R-CNN offers high accuracy, end-to-end training, and shared feature extraction advantages that render it a widely adopted target detection tool. Hence, this paper adopts Faster R-CNN as the fundamental network architecture. ResNet 50 is a renowned feature extraction network that effectively tackles gradient disappearance and explosion issues in deep networks through its residual structures. It also possesses profound network layers and robust initial feature extraction capabilities, making it an optimal choice for deployment as the feature extractor for Faster R-CNN. The amalgamation of these two models significantly enhances target detection accuracy and performance, thereby establishing them as prevalent options for target detection tasks. This paper proposes a deep learning network based on the framework of Faster R-CNN + ResNet 50. However, due to the diverse application scenarios and variations in target types for detection, the fundamental network framework may not achieve optimal accuracy in terms of detection. For instance, given the significant size disparity of aluminum defects, a single output feature layer cannot encompass all dimensional defects adequately. Consequently, the basic network structure meets industrial requirements concerning the effective discrimination and accurate detection of aluminum defects.

**Figure 1:**
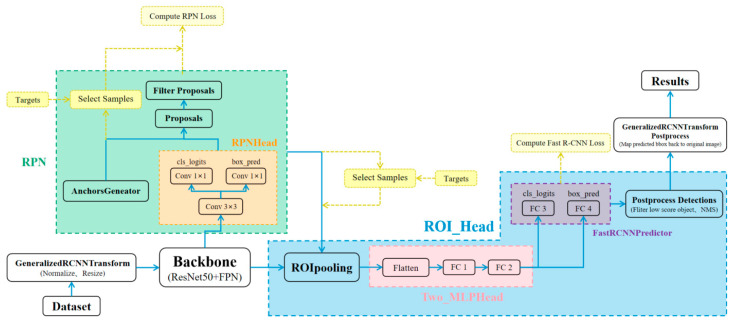
The proposed comprehensive architecture of the deep learning network.

The feature pyramid (FPN) structure is commonly employed to address the fusion of multi-scale information in tasks such as object detection and semantic segmentation, with the aim of enhancing the feature representation capability of deep convolutional neural networks across different scales. In the Faster R-CNN network, the ROI-Pooling algorithm is utilized to extract the output feature map from the feature extraction network; however, this process involves two quantization steps that can potentially reduce both precision and accuracy in terms of network detection. To overcome this limitation, we introduce the ROI-Align algorithm [17], which is a technique used in object detection to improve the precision of feature extraction by precisely mapping region proposals to feature maps. It eliminates the double-line interpolation used in ROI-Pooling to avoid quantization processing and maintain high detection accuracy. Moreover, to ensure the accurate identification of defects at various sizes and improve overall detection accuracy, as shown in Figure 1, we incorporated the FPN structure and employed the genetic K-means clustering algorithm to obtain a global optimal solution for prediction boxes within our enhanced network framework. This was achieved by combining the global search capability of genetic algorithms with the rapid convergence of K-means clustering. Initially, multiple sets of clustering centers are generated as the population. The genetic algorithm evaluates and selects individuals based on fitness, which is determined by the clustering loss (e.g., within-cluster sum of distances). Through iterative crossover, mutation, and K-means optimization steps, the algorithm ensures that prediction boxes converge to a globally optimal solution, balancing precision and computation efficiency.

### 2.2. Improved Network’s Optimized CBAM

Improvements in network infrastructure can effectively enhance the accuracy and universality of network detection. The identification of aluminum defects poses challenges due to their small size and subtle differences. To better suit the detection and identification of such defects, it is necessary to further improve the feature extraction capability of the network. A Convolutional Block Attention Module (CBAM) is an attention mechanism used to enhance target detection performance in convolution neural networks by reducing dimensionality through whole-connection sharing layer characteristic figures, thereby decreasing parameter numbers while assigning corresponding weights to each channel value [18]. However, this process may have an adverse effect on channel dependence as fully connected layers tile all feature layers into one-dimensional vectors before giving them corresponding weights for dimensionality reduction. Therefore, this paper replaces and optimizes the full-connection layer in CBAM.

**Figure 2:**
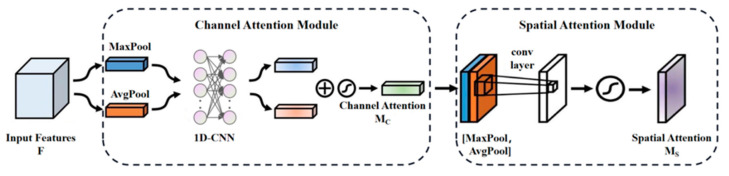
Optimized CBAM schematic diagram.

To ensure inter-layer dependency and minimize the number of parameters and computations, this study replaces two fully connected layers with a one-dimensional convolution layer. By applying one-dimensional convolution at the image channel level, it guarantees correlation between feature layers while reducing computational complexity compared to two-dimensional convolution layers. To incorporate channel attention, different one-dimensional convolution methods are employed for the MaxPool and AvgPool output feature maps, followed by their superposition in order to achieve an optimized CBAM, as depicted in Figure 2.

The convolution kernel size can be adaptively generated to accommodate different input image sizes. In this paper, the Efficient Channel Attention (ECA) network’s adaptive convolution kernel is referenced [19]. The adaptive convolution kernel of the ECA network determines the size of the one-dimensional convolution kernel based on the number of channels in the input feature map. The specific calculation formula is presented in Equation (1). The enhanced CBAM mixed-attention mechanism is incorporated into the convolutional module of the ResNet 50 feature extraction network to enhance the deep learning networks’ capability in terms of extracting features.

(1) $$T=\left|\frac{\log_{2}\left(c\right)+b}{\gamma }\right|$$

where *T* represents the size of the one-dimensional convolution kernel, *c* represents the properties of the input properties, and *b* and *γ* represent the corresponding hyperparameters.

### 2.3. The Framework of the Lightweight Deep Learning Detection Network

To fulfill the requirements of industrial inspection, the detection network must possess not only high accuracy but also efficient detection speed. Given the large number of parameters and complex network structure, it becomes imperative to lightweight the network for improved detection efficiency and reduced time consumption. In 2020, Huawei Noah’s Ark Laboratory proposed a novel unit called a Ghost model as a fundamental component of deep learning networks, distinct from traditional convolutional layer units. The Ghost model employs a limited number of convolutional parameters to generate multiple feature maps, thereby reducing both the model parameter count and size. The output layer of the Ghost model consists of two parts: one part represents feature maps obtained through conventional convolutions on input layers, while the other part comprises feature maps derived from cost-effective operations applied to previous sections. Consequently, this latter set can be regarded as ‘ghosts’ representing earlier stages in terms of their features.

Assuming that Din denotes the number of channels in input feature graphs, Dout signifies the channel count in the output layers, *k* represents the kernel size used during the traditional convolution process, *r* indicates the ratio between the actual feature graphs and the phantom ones generated by these graphs (with *d* denoting the kernel size for the phantom feature graph), we calculate the parameter count for Ghost models using Formula (2).

(2) $$S_{Ghost}=D_{in}\times k^{2}\times D_{out}+\left(1-r\right)\times D_{out}\times d^{2}$$

Compared with traditional convolution, the parameter reduction in the Ghost model can be reformulated as follows:

(3) $$\Delta S=D_{in}^{\prime }\times k^{2}\times D_{out}^{\prime }-\left[D_{in}\times k^{2}\times D_{out}+\left(1-r\right)\times D_{out}\times d^{2}\right]$$

**Figure 3:**
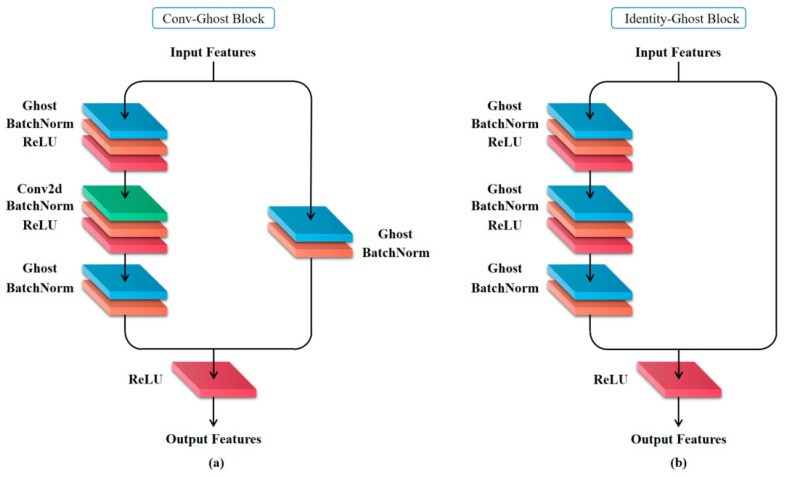
The schematic diagram illustrates the states of two module replacements: (**a**) Conv–Ghost Block; (**b**) Identity–Ghost Block.

In this study, the ratio between the feature map and Ghost feature is at 1:1 (i.e., *r* = 0.5), while the volume kernel size of the Ghost feature map is fixed. Figure 3 illustrates the replacement of convolutional layers in both types of blocks within ResNet 50 with our proposed Ghost model. This approach solely modifies the volumetric layer structure in ResNet 50, without altering the specific configurations of Conv Block and Identity Block. The enhanced Conv Block and Identity Block collectively form a ResNet 50—ours network is based on its original architecture.

## 3. Experimental Preparation

### 3.1. Dataset

**Figure 4:**
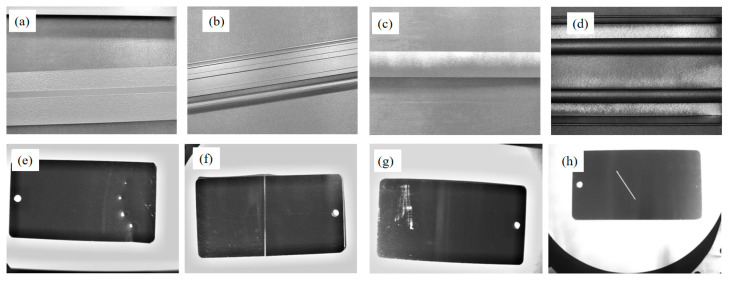
Sample images of different defects: (**a**) under screen; (**b**) crocodile skin; (**c**) non-conducting; (**d**) corner leakage; (**e**) crater; (**f**) angle hole; (**g**) scrape; (**h**) scratch.

This study selects common types of defects in the production and use of aluminum materials. The dataset is divided into publicly available datasets and defect datasets simulated through experimental methods. The publicly available datasets include five types of defects: variegated, crocodile skin, under screen, corner leakage, and non-conducting, which are displayed in Figure 4. The simulated defect types include four types: angle hole, crater, scratch, and scrape. The dataset comprises 3200 images, with a training-to-validation ratio of 8:1. Specifically, the final training set consists of 2560 images, while the validation set includes 320 images and the test set contains 320 images.

### 3.2. Image Sensor-Equipped Acquisition System

**Figure 5:**
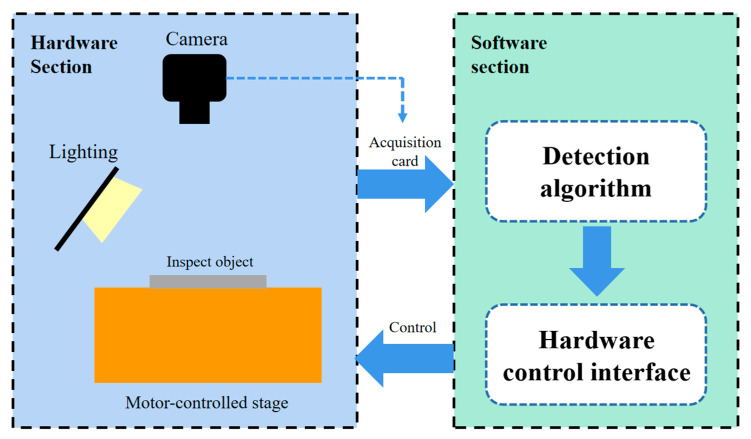
Detection System Structure Diagram.

This study’s acquisition system can be divided into three parts: the hardware acquisition section, the software detection section, and the connection section. Each module performs different functions. Figure 5 shows the overall structure of the detection system.

The hardware acquisition section is responsible for image acquisition. The object to be inspected is placed on a motor-controlled stage and moved into the camera’s field of view. The camera, equipped with an image sensor using a CMOS photosensitive chip, is used to capture images. Appropriate lighting and light arrangement are applied to create the desired light field, and the camera’s focal length is adjusted before capturing the image.

The connection section primarily functions to link the hardware and software components. First, the camera transmits the captured image to the image acquisition card. The computer then receives the image data from the acquisition card, preprocesses it, and finally transmits the image to the detection algorithm. The connection section is not only responsible for transferring image data but also for receiving control signals. The system software sends control instructions to the microcontroller, which then controls the motor movement, allowing the software to control the hardware.

### 3.3. Experimental Environment

The training and detection experiments of the deep learning network were conducted in a Windows environment, with the details provided in Table 1. The experiments primarily aimed to validate the detection accuracy, speed, model size, and optimized network stability. Detection accuracy was evaluated using mean Average Precision (mAP) under various intersection-over-union (IOU) conditions, while detection speed was measured by analyzing the time taken to detect 100 images. Additionally, the model’s loss function was utilized to assess both model stability and network feature extraction capability.

## 4. Results and Discussion

### 4.1. Comparison of Loss Functions for Training and Validation

**Figure 6:**
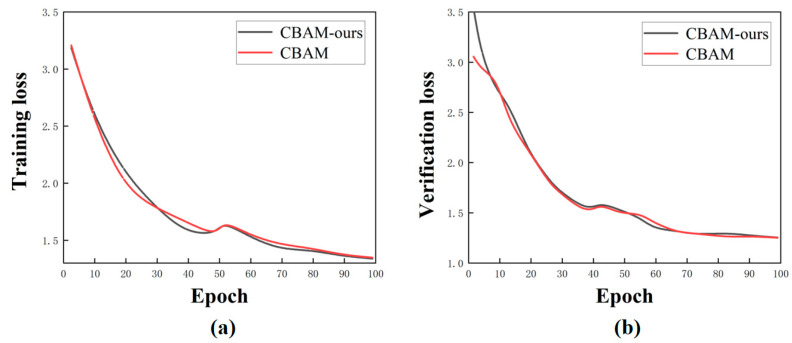
Comparison of loss functions of the attention module before and after enhancement. (**a**) Loss function on the training set; (**b**) loss function on the validation set.

The training process was conducted within the aforementioned experimental environment. Figure 6 illustrates a comparison of loss functions between the training and validation sets before and after implementing CBAM improvements. Figure 6a presents the loss function comparison for the training set, whereas Figure 6b displays the loss function comparison for the validation set.

**Figure 7:**
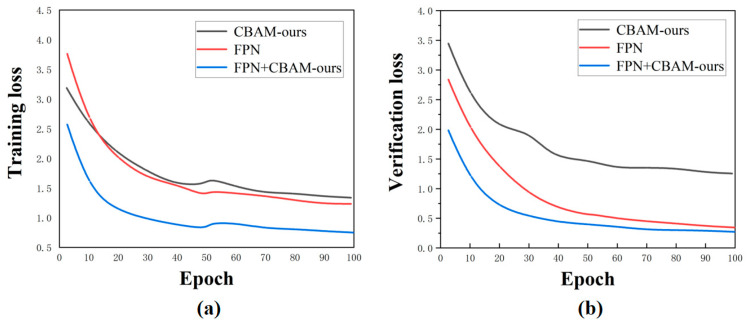
Comparison of the loss functions of the network under different structures: (**a**) training set loss function; (**b**) validation set loss function.

The loss function characterizes the feature extraction capability of the feature extraction network in the detection network. As depicted in Figure 6a, the training loss function of the attention mechanism integrated into the ResNet 50 network exhibits a significant reduction after improvement, indicating a substantial enhancement in the feature extraction capability of the network following module addition. In Figure 6b, it can be observed that, compared to changes in the validation set loss function before and after incorporating the attention module into ResNet 50, noise influence is reduced post the addition of the attention module, rendering the network more robust and stable. Additionally, there is a similar trend between pre- and post-improvement loss functions of the attention module, suggesting reasonable optimization of the original attention mechanism to some extent. To validate the overall stability of our proposed network architecture, we combined both the attention module and the feature pyramid with the Faster R-CNN structure under identical training conditions using the same set for training purposes. Furthermore, we compared the results obtained by individually adding either the attention module or the feature pyramid to those achieved when both structures were incorporated into our network configuration separately. Figure 7 illustrates the loss function of an improved CBAM alone, a single FPN alone, as well as their interaction with the Faster R-CNN framework, respectively.

In Figure 7a, the training loss function of the two together is significantly smaller than that of each individual component, indicating their prominent roles in the feature extraction process. It should be noted that the proposed module emphasizes importance within layers and across different positions, while the FPN focuses on information fusion and convolutional information preservation. Together, they enhance the feature extraction capability of the network, resulting in a much lower training loss function compared to using either component alone. In Figure 7b, when the FPN operates independently, its validation loss function is closer to that when it operates jointly with other components. However, this mechanism exhibits a larger loss function due to its output being limited to a single feature layer which leads to poor generalization performance. On the other hand, the FPN produces four output feature layers, from large to small sizes, enhancing its robustness. The detection network inherits this generalization ability from the FPN and consequently achieves a smaller validation loss function.

### 4.2. Impact of Diverse Attention Mechanisms on Detection Accuracy

Mean Average Precision (mAP) serves as a crucial metric that reflects the overall accuracy of the detection network by evaluating both precision and recall across different classes and intersection-over-union (IOU) thresholds. mAP is calculated as the mean of the Average Precision (AP) values for each class, where AP is computed by integrating the precision–recall curve. The AP for each class is given by the area under its precision–recall curve, which measures how well the model detects objects in that class across varying confidence thresholds.

To compute mAP, the IOU threshold is used to determine whether a detected bounding box is considered a “true positive”. The IOU between the predicted bounding box (*B_p_*) and the ground truth box (*B_gt_*) is calculated as:

(4) $$\mathrm{IOU}=\left|\frac{B_{p}\cap B_{gt}}{B_{p}\cup B_{gt}}\right|$$

where |*B_p_*∩*B_gt_*| is the area of overlap between the predicted and ground truth boxes, and |*B_p_*∪*B_gt_*| is the area of their union.

The AP for each class can be defined as the average of precision values at various recall levels, typically integrated across a range of recall points. The mAP is then the mean of these AP values across all classes:

(5) $$\mathrm{mAP}=\frac{1}{N}\sum_{i=1}^{N}{\mathrm{AP}}_{i}$$

where *N* is the total number of classes, and AP*_i_* is the Average Precision for class *i*.

In this study, we compare the variations in mAP values with different IOU thresholds. Table 2 presents the mAP values of ResNet 50 after incorporating two attention modules at various IOU levels, where 0.5 represents the mAP value when IOU is set to 0.5. A higher IOU value indicates better alignment between model-generated bounding boxes and actual targets, making it more stringent in determining true detection. The results demonstrate a significant decrease in mAP as the IOU threshold increases. Notably, the mAP values of our optimized CBAM are higher than those of its original counterpart as the IOU threshold increases. The experimental evaluations further confirm that our improved CBAM outperforms the unimproved version in terms of precision and recall rates.

**Figure 8:**
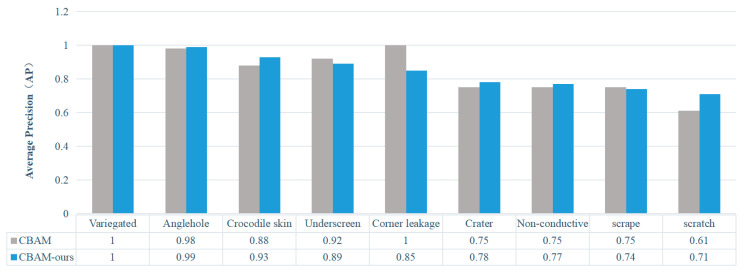
mAP of the detection of different defects between a conventional CBAM and our optimized CBAM.

The comparison of detection accuracy for different defects using conventional and optimized attention modules is illustrated in Figure 8. It can be observed that the optimized attention module effectively enhances the detection accuracy for most defects, particularly improving the accuracy for scratch defects. However, a decrease in detection accuracy is observed for corner leakage, under screen and scrape defects. This study suggests that this may be attributed to the small size of the leakage angle defect, resulting in a small detection frame. Consequently, with the addition of one-dimensional convolution, the detection frame is neglected and information from fully connected layers is lost during the dimensionality reduction process, leading to reduced detection accuracy.

To assess the overall accuracy of the proposed network in this study, we further compared the mAP values of the enhanced attention module and feature pyramid under different IOU conditions when they are utilized individually and when integrated into the network. As presented in Table 3, for mAP0.5, there is only a marginal 0.84% difference between the FPN and the FPN combined with our optimized attention mechanism. Since improving higher mAP values becomes progressively more challenging, enhancements are less pronounced at an IOU condition of 0.5. However, as IOU increases, accentuating important regions by means of attention modules becomes more prominent, resulting in a significantly larger disparity between FPNs and those combined with an attention mechanism.

**Figure 9:**
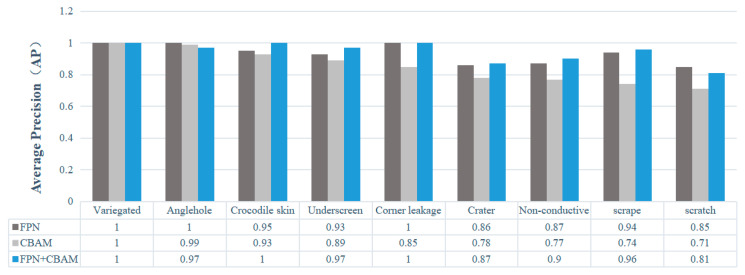
mAP values for the detection of different defects across different network frameworks.

The mAP values of various defect detection results by network, under a single-FPN structure and in combination with an attention module, are illustrated in Figure 9. It can be observed that the test accuracy of the two factors combined generally surpasses that of the single-factor network. The attention mechanism assigns defect regions with varying lengths and widths to larger feature layers during detection, leading to a decrease in detection accuracy. Moreover, compared to a network with only a single multi-scale feature pyramid, scratch defects exhibit higher detection accuracy. This may be attributed to their smaller size but larger prediction frame, causing less focus to be placed on scratch defect features within the attention mechanism and resulting in a lower detection accuracy. For most defects, incorporating both an attention module and multi-scale pyramid into the network structure yields higher detection accuracies than with either component included separately.

### 4.3. Effect of Model Size on Detection Efficiency

The model size, training speed, and detection speed of ResNet 50 with the attention module before and after improvement are presented in Table 4. It can be observed that the addition of the attention module increases the model size. However, a reduction of approximately 14 M in overall network model space is achieved through lightweight processing. Notably, in addition to reducing the model size, our proposed approach also enhances both the training and detection speeds compared to its counterparts prior to enhancement, due to fewer steps being involved in obtaining channel attention using the one-dimensional convolution. The mAP value of the modified ResNet 50 network (i.e., ResNet 50 + FPN + CBAM-ours) after lightweight processing achieves a remarkable accuracy rate of 93.77%. Moreover, the improved model exhibits faster training and detection speeds compared to its counterpart, while also reducing the model size by 14.3%. Consequently, our approach ensures accurate detection by the model while simultaneously minimizing space requirements and enhancing overall performance in line with industry standards.

### 4.4. Demo of Detection Results with Proposed Deep Learning Network

**Figure 10:**
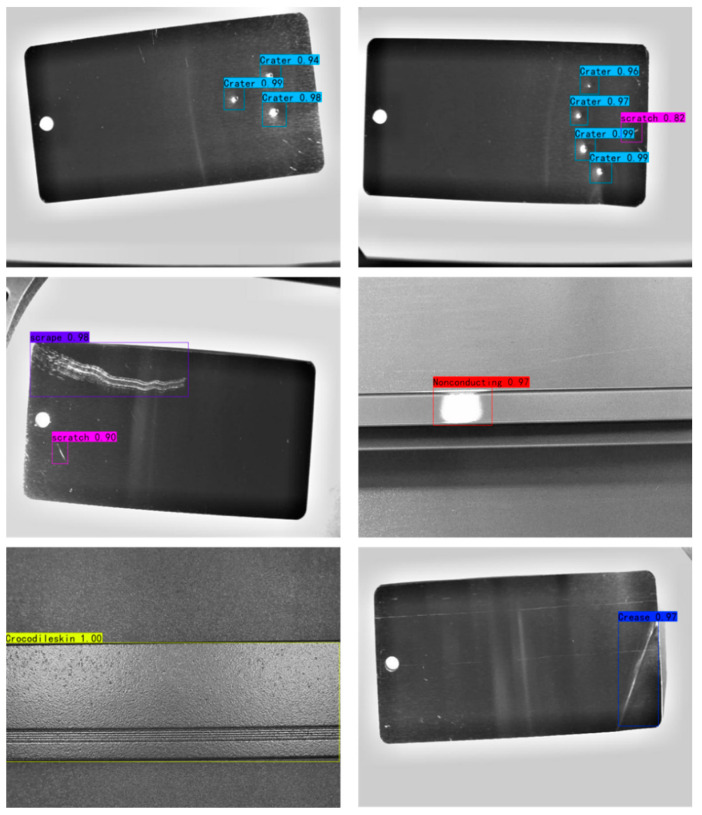
An exemplification of detection outcomes utilizing an optimized Faster R-CNN network, where distinctively colored bounding boxes indicate defect types along with their corresponding confidence scores.

The results of aluminum defect detection using the proposed deep learning network are presented in Figure 10, illustrating the detection outcomes for various defect types. It is evident that no false or missing detections occur, and there is negligible deviation in the predicted box positions. Both larger and smaller defects can be accurately identified in terms of their type and location. These experimental results demonstrate that the optimized Faster R-CNN network yields excellent performance in detecting aluminum defects within industrial settings.

## 5. Conclusions

In summary, a novel aluminum defect detection network based on the optimized Faster R-CNN architecture is presented and comprehensively analyzed. The main conclusions are as follows:

(1) To enhance the information utilization rate between features and convolutions, a multi-scale feature pyramid was incorporated into the feature extraction network. Additionally, the ROI-Pooling algorithm was replaced with the more accurate ROI-Align algorithm to prevent any loss in network accuracy.

(2) An optimized attention module based on CBAM was employed to improve both convolutional efficiency and information utilization. This module not only reduces model parameters but also enhances training speed as well as the detection speed of the network, resulting in an achieved mAP value of 94.25%.

(3) The Ghost model replaced convolution blocks within the deep learning network, effectively reducing model parameters by 14.3%. Consequently, this lightweight design achieved a detection accuracy of 93.77%, fulfilling industrial detection requirements.

## Figures and Tables

**Table 1:** Experimental environment.

Parameter	Parameter Value
Operating system	Windows 10
CPU model	Intel(R) Core(TM) i9-10980xE CPU @ 3.00 GHz
Tensoflow version	2.7.0
Python version	3.7
Initial learning rate	1 × 10^−4^
Iterations	100

**Table 2:** mAP values for different models.

Network Model	mAP (0.5)	mAP (0.65)	mAP (0.75)
ResNet 50	60.0%	46.28%	33.0%
ResNet 50-CBAM	84.76%	78.45%	70.48%
ResNet 50-CBAM-ours	85.16%	79.39%	70.57%

**Table 3:** Comparison of the mAP values of various detection networks with different modules.

Network Model	mAP (0.5)	mAP (0.65)	mAP (0.75)
CBAM-ours	85.16%	79.39%	70.57%
FPN	93.41%	88.85%	82.23%
CBAM-ours + FPN	94.25%	91.30%	84.18%

**Table 4:** Different model sizes and detection speeds.

Network Model	Model Size (KB)	Training Speed (min/epoch)	Detection Speed (Frame per Second, FPS)
ResNet 50	101,472	9	4.4
ResNet 50-CBAM	113,017	12	4.11
ResNet 50-CBAM-ours	86,914	11	4.19

## Data Availability

The data presented in this study are available on request from the corresponding author. The data are not publicly available due to privacy issues.
